# Molecular Structural Analysis of Porcine CMAH–Native Ligand Complex and High Throughput Virtual Screening to Identify Novel Inhibitors

**DOI:** 10.3390/pathogens12050684

**Published:** 2023-05-05

**Authors:** Oluwamayowa Joshua Ogun, Georg Thaller, Doreen Becker

**Affiliations:** 1 Institute of Animal Breeding and Husbandry, University of Kiel, Olshausenstraße 40, 24098 Kiel, Germany; 2 Institute of Genome Biology, Research Institute for Farm Animal Biology (FBN), Wilhelm-Stahl-Allee 2, 18196 Dummerstorf, Germany

**Keywords:** sialic acid, Neu5Gc, Neu5Ac, CMAH, pathogenesis, Alphafold2, Vina, inhibitors

## Abstract

Porcine meat is the most consumed red meat worldwide. Pigs are also vital tools in biological and medical research. However, xenoreactivity between porcine’s N-glycolylneuraminic acid (Neu5Gc) and human anti-Neu5Gc antibodies poses a significant challenge. On the one hand, dietary Neu5Gc intake has been connected to particular human disorders. On the other hand, some pathogens connected to pig diseases have a preference for Neu5Gc. The Cytidine monophospho-N-acetylneuraminic acid hydroxylase (CMAH) catalyses the conversion of N-acetylneuraminic acid (Neu5Ac) to Neu5Gc. In this study, we predicted the tertiary structure of CMAH, performed molecular docking, and analysed the protein–native ligand complex. We performed a virtual screening from a drug library of 5M compounds and selected the two top inhibitors with Vina scores of −9.9 kcal/mol for inhibitor 1 and −9.4 kcal/mol for inhibitor 2. We further analysed their pharmacokinetic and pharmacophoric properties. We conducted stability analyses of the complexes with molecular dynamic simulations of 200 ns and binding free energy calculations. The overall analyses revealed the inhibitors’ stable binding, which was further validated by the MMGBSA studies. In conclusion, this result may pave the way for future studies to determine how to inhibit CMAH activities. Further in vitro studies can provide in-depth insight into these compounds’ therapeutic potential.

## 1. Introduction

Porcine meat, one of the primary animal protein sources, is the most popular red meat consumed worldwide [1]. Additionally, pigs share a significant number of physiological and anatomical features with humans and are vital tools in biological and medical research [2,3]. Pig organs are also used as replacements for failed human organs in cross-species transplantation (xenotransplantation) [4]. Despite the significance of everything mentioned above, one major challenge of porcine meat consumption and xenotransplantation is the xenoreactivity between the N-glycolylneuraminic acid (Neu5Gc) of porcine meat/organs, a sialic acid (Sia), and the human anti-Neu5Gc antibodies. These xenoreactivities are found to be associated with specific diseases and disorders, such as cancer and diabetes [5]. Neu5Gc is absent in poultry meat and fish, but high contents are found in pork and other red meat products [6].

The common Sias in mammals are Neu5Gc and N-acetylneuraminic acid (Neu5Ac). The enzyme Cytidine monophospho-N-acetylneuraminic acid hydroxylase (CMAH) encoded by the *CMAH* gene catalyses the conversion of the activated Neu5Ac (CMP-Neu5Ac, i.e., the native substrate or ligand of CMAH) to Neu5Gc. Only Neu5Ac is present in humans due to a mutation in the human *CMAH* that leads to gene inactivation [7,8]. 

Sia can exhibit structural diversity generated by various linkages from its 2-carbon to underlying glycans. These Sia-linkages are typically formed at the 3- or 6-position of galactose (Gal) residues or the 6-position of N-Acetylgalactosamine (GalNAc) residues [9]. Pigs express the α2-3 Sia-linkage (avian type) and the α2-6 Sia-linkage (preferred human type), as well as the two primary Sia structures (Neu5Gc and Neu5Ac). Sias are known receptors for a variety of viruses and bacteria. Studies have demonstrated that there is a high affinity between specific bacteria and viruses and Neu5Gc [10,11,12]. The resultant diseases of this affinity with porcine Neu5Gc could have an unpredictable economic impact on pig breeding. Pigs are also referred to as “mixing vessels” of influenza viruses between avian species and humans [13]. Gene exchange between avian, swine, and human viruses can occur in pigs [14,15], creating a novel virus that could cause human pandemics [16].

The challenges posed by the presence of Neu5Gc in pig cells cannot be simply dismissed. The functional and structural studies of the porcine CMAH enzyme remain scarce due to the unavailability of the tertiary structure of the protein in the databases, thereby limiting studies on how this enzyme is regulated via different interactions, such as ligand(s) and other protein(s). In general, structural predictions of proteins have been used to identify protein families, significant functional groups, and protein interactions [17]. For instance, the resolved tertiary structure of the SAR-CoV2 protein has been used in studies to discover critical binding sites, distinct interactions with inhibitors [18,19], and the impact of mutations [20,21] to gain a deeper understanding of the virus. 

**Figure 1:**
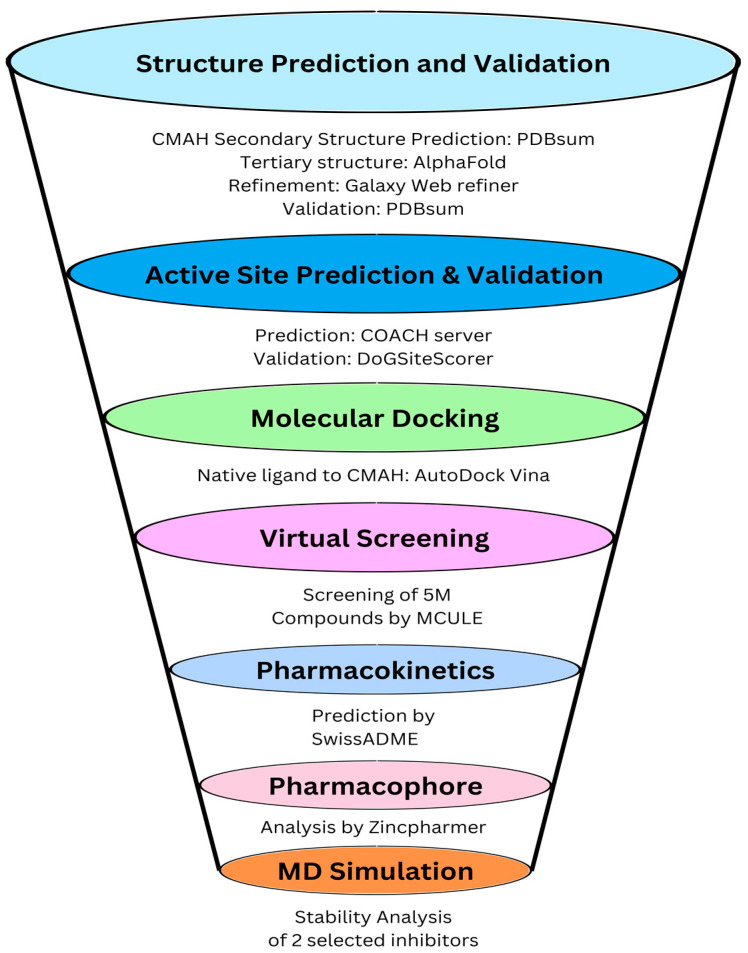
Schematic workflow of the molecular structural analysis of porcine CMAH–native ligand complex and virtual screening to identify novel inhibitors.

Several groups have eliminated Neu5Gc in pig cells using genome editing approaches [22,23,24,25,26]. Although, most of those studies performed the elimination primarily for reasons related to xenotransplantation rather than for dietary purposes. The ethical argument regarding genome editing and its sometimes irreversible consequences on the organism (off-target effects) is also challenging. In this study, we employed a different approach to genome editing, using molecular structural information with the help of various computational tools as a potential solution to prevent or inhibit the biosynthesis of Neu5Gc in porcine cells by identifying potential competitive inhibitors of the native ligand of CMAH. The tertiary structure of the CMAH protein was predicted with deep learning tools. High throughput structure-based virtual screening and computational physicochemical properties analysis of the novel inhibitors were performed. The binding modes of the screened inhibitors were initially predicted by molecular docking. Molecular docking helps to predict the optimal orientation of a ligand in the binding pocket of a target protein. The highest Vina or negative scores could be viewed as signs of more effective binding interactions, which could be used as basis for the selection of the top or best inhibitors [27,28,29,30,31]. Although, ranking based on Vina scores are mostly effective for predicting binding poses rather than the effectiveness of the binding affinity [32,33]; however, further in-depth molecular dynamics (MD) simulations or validations with experimental data are usually required. In addition to this study, the complexes were subjected to molecular dynamics simulation to assess their stabilities. MD simulation helps to analyse the ligand and protein flexibility. The main advantage of this process is that it mimics the physical environment, that is, how the protein and ligands interact with each other in time. It also helps in analysing the flexibility of structures and their entropic effects [34]. A flowchart of the study is shown in Figure 1.

## 2. Materials and Methods

In this study, we employed computational approaches to determine the CMAH protein structure and its inhibitors. The ab initio approach was employed for the structure prediction, and validation was performed through several other tools. The inhibitors of this protein were first virtually screened, and the obtained two top inhibitors were molecularly docked and simulated for their interaction affinity and stability assessment.

### 2.1. Prediction, Refinement, and Validation of Tertiary Structure of CMAH Protein

The secondary structure of CMAH was predicted through the European Molecular Biology Laboratory-European Bioinformatics Institute (EMBL-EBI) tool, PDBsum (http://www.ebi.ac.uk/thornton-srv/databases/pdbsum/, accessed on 1 May 2023) [35]). The absence of the tertiary structure of CMAH in the database is hampering further stud ies on how this protein interacts with ligands and other proteins at the molecular level. Data, such as the binding site residues, are also not available. We predicted the tertiary structure using AlphaFold2 (ColabFold). The AlphaFold2, a deep learning approach, was regarded as an effective method following its breakthrough in 2021 [36]. The collaboration between ColabFold and Google made the open-source platform software available at https://github.com/sokrypton/ColabFold (accessed on 1 May 2023). Protein structures are predicted by generating sequence alignments through MMseqs and HHsearch [37].

The CMAH protein sequence (NP_001106486.1) was retrieved from the NCBI protein database (https://www.ncbi.nlm.nih.gov/, accessed on 1 May 2023). We performed additional protein structure refinement using the GalaxyWEB refiner tool (https://galaxy.seoklab.org/index, accessed on 1 May 2023). The server uses MD simulations to execute repetitive structure perturbation and eventual overall structural relaxation [38]. The tertiary structure was validated using the PDBsum webserver (http://www.ebi.ac.uk/thornton-srv/databases/pdbsum/Generate.html, accessed on accessed on 1 May 2023) to analyse the Ramachandran plot [35] and ProSA online tool (https://prosa.services.came.sbg.ac.at/prosa.php, accessed on accessed on 1 May 2023) for additional analysis [39]. We also incorporated ERRAT analysis to validate the predicted structure’s accuracy [40].

### 2.2. Prediction of Active Site Residues

The protein’s active site was predicted by the COACH online tool (https://zhanggroup.org/COACH/, accessed on 1 May 2023). It employs a meta-server approach to generate predictions for complementary ligand binding sites based on the structure of target proteins using two comparative methods: TMSITE and SSITE. The COACH compares binding-specific substructures and sequence profiles to recognise active sites of templates from the BioLiP protein function database [41,42]. It combines the outcomes of the other three cutting-edge techniques: COFACTOR, FINDSITE, and ConCavity [42]. Users can either input primary sequences or tertiary structures. The result was cross-validated with the DoGSiteScorer tool from the protein plus online tool (https://proteins.plus/, accessed on 1 May 2023). This tool is a “grid-based method that utilises a difference of Gaussian filter to identify potential binding pockets based solely on the tertiary structure of the protein and then divides them into sub-pockets” [43].

### 2.3. Computational Docking of Porcine CMAH and the Native Ligand

The three-dimensional structure of the native ligand CMP-Neu5Ac was downloaded from the PubChem database (CID 448209; https://pubchem.ncbi.nlm.nih.gov/, accessed on 1 May 2023) [44]. The OpenBabel software version 3.1.1 was used to convert the ligand structure-data file (SDF) format to Protein Data Bank (PDB), [partial charge (Q), and atom type (T)] (PDBQT) format and prepared for docking. All rotatable bonds were kept flexible. Additionally, the tertiary structure of CMAH was uploaded into the molecular graphic laboratory (MGL) software version 1.5.7 and prepared for docking by adding polar hydrogen and Kollman charges. The grid box for the docking calculation was centred on the protein’s active site (X = 2.074, Y = 1.004, Z = −0.609) and size (X = 95, Y = 95, Z = 95), encompassing all the amino acid residues. The configuration file defined the coordinates of exhaustiveness and energy ranges as 80 and 4, respectively. The prepared structure was saved in the PDBQT file format for molecular docking using the AutoDock Vina application of the MGL software. After docking, the MGL tool, Discovery studio, protein plus (https://proteins.plus/, accessed on 1 May 2023) [45], and protein–ligand interaction profiler (PLIP; https://plip-tool.biotec.tu-dresden.de/plip-web/plip/index, accessed on 1 May 2023) online tools [46] were used to visualise and analyse the AutoDock Vina output file, and the best conformation was selected.

### 2.4. High Throughput Structure-Based-Virtual Screening

Structure-based-virtual screening (SBVS) is a computational approach that efficiently identifies key inhibitors or lead compounds from many compounds based on interactions within the active or binding pockets of target proteins [47]. The SBVS was performed with MCULE (https://mcule.com/, accessed on 1 May 2023), an online database with millions of synthetically accessible molecules. Fast results are possible due to many the central processing units (CPUs) of cloud machines [48].

The tertiary protein structure of CMAH was uploaded in the PDB format. The basic Lipinski’s rule of five [49], otherwise known as Pfizer’s rule of five, was used as the property filter in the workflow of the SBVS; that is, hydrogen bond donor (HBD) not more than 5, hydrogen bond acceptor (HBA) not more than 10, molecular mass less than 500 Daltons, and log P (octanol-water partition coefficient) not more than 5. In addition, the polar surface area was adjusted to 140 Å^2^ maximum, sampler size to 1000, and similarity cut off at 0.7 and 3 million as the maximum number of compounds after sphere exclusion. Other settings of the MCULE were kept at default. An estimated 5,000,000 ligands were screened against the CMAH active site. Lastly, the same grid box calculated above for the molecular docking of CMAH and the native ligand was used. 

The virtual screening was performed with the inbuilt Vina tool. The top two inhibitors having the highest Vina-docking score were selected as a basis for further validations. The selected inhibitors were also assessed for their pharmacokinetic properties and compared with the native ligand through the SwissADME online tool (http://www.swissadme.ch/, accessed on 1 May 2023) [50]. The absorption, distribution, metabolism, and excretion (ADME) analytic tools are customised for humans. Since pigs are used in human preclinical trials [51,52,53], we included the analysis to provide insight into the inhibitors’ applicability at the clinical level. In addition, the physicochemical properties and the amino acid residues interacting with the target protein were assessed. The pharmacophore analyses were performed through the Zincpharmer online tool (http://zincpharmer.csb.pitt.edu/ accessed on 1 May 2023) [54]. The inhibitors were loaded, and pharmacophore features were displayed after utilising the load features option. The images obtained were saved for further analysis.

The complexes were further visualised and analysed with tools such as Discovery studio, protein plus, and PLIP online tools. Furthermore, to assess the accuracy of the molecular docking, the catalytic domain of CMAH was particularly docked with sorted inhibitors and analysed for the similarity between docked complexes consisting of apo-CMAH-inhibitors and catalytic domain-CMAH.

### 2.5. Molecular Dynamic Simulations of the Complexes

The complexes were subjected to a 200 ns MD simulation. CHARMM-GUI server was used to prepare the input files for simulation. The Antechamber program embedded in the server generated the charm topology and ligand parameter files [55]. Subsequently, the solvation of complexes was performed in a periodic box of size 10 Å containing TIP3P water molecules [56], then neutralised by adding Na^+^ and Cl^−^ ions. After neutralisation, the steric clashes were removed via minimisation of the systems for 5000 steps that followed the equilibration steps in NVT and NPT ensembles for 50,000 and 100,000 steps, respectively. Finally, the systems were subjected to a production run at 310K temperature [57]. The constant temperature (310 K) and pressure (1 atm) were maintained by the Berendsen thermostat and Parrinello–Rahman algorithms. The LINCS algorithm [58] was applied to constrain the optimal lengths of hydrogen atoms, while non-bonded interactions were dealt with using the Verlet algorithm [59]. The short-range electrostatic interactions were computed using the Particle Mesh Ewald method [60]. The CHARMM36 forcefield [61] was used during the production run. The MD trajectories were stored at every 10 ps and then analysed using gmx rms, gmx_rmsf, gmx_area, gmx_cod, and gmx_gyrate commands. VMD and PyMOL were used to investigate the hydrogen bonds [62].

## 3. Results

### 3.1. Predicted Active Site Residues and Tertiary Structure Validation

Before proceeding to tertiary structure prediction, the secondary structure of the CMAH protein was predicted. Secondary structure analysis indicated that beta-sheets dominate the CMAH structural configuration, and most residues at N-terminus participate in beta-sheets formation (Figure S1).

**Figure 2:**
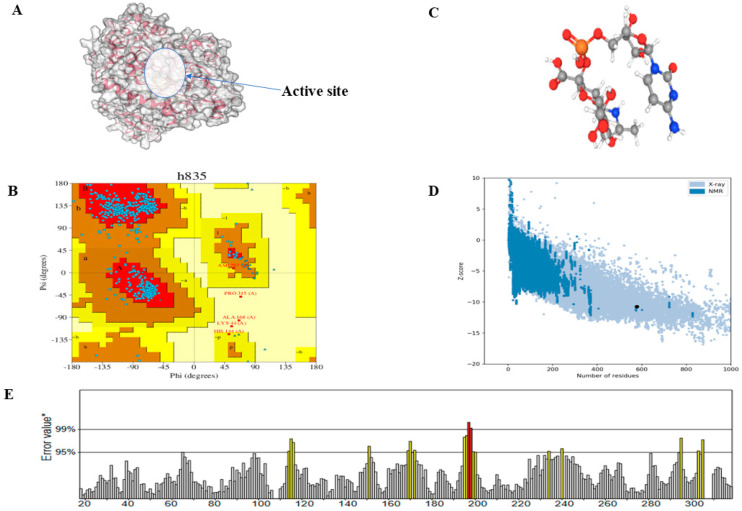
Visualisation of the tertiary structure of the CMAH protein with the active site obtained via protein plus tool (**A**). Ramachandran’s plot depicted that 91.4% of the amino acid residues are located in the most favoured regions (**B**). The 3D structure of CMP-Neu5Ac was extracted from the PubChem database (**C**). The ProSa analysis image shows a Z-score of −10.73 (**D**). ERRAT overall score indicates a 95% quality factor for the predicted structure. Yellow colour indicates the region that can be rejected at the 95% confidence level (intermediate quality or disordered region). Regions that can be rejected at the 99% level are shown in red (low quality) (**E**).

In drug discovery, the validation of predicted protein structures is essential. The AlphaFold2 performed the initial step of the structural prediction, and refinement of the structure was further carried out by the GalaxyWEB server refiner tool [38]. The PDBsum, a web-based application, was used to analyse the structural information and quality of the protein’s tertiary structure. The PDBsum results are image-based and analyses of the tertiary structure quality were performed using the PROCHECK tool [35]. The Ramachandran plot analysis of the tertiary structure revealed that 91.4% of the residues were found in the most favoured regions (Figure 2B). The result from ProSa revealed that the predicted protein has a Z-score of −10.73. This shows that the structure conforms to the standard X-ray crystallography for proteins of a similar size (Figure 2D). ERRAT analysis also gave the overall quality factor of 95.007%, which is indicative of a high-quality protein structure (Figure 2E). The overall analysis of this protein indicates that the protein is of high quality and may be used for structure-based drug discovery.

The result from the COACH server predicted 10 amino acid residues as the active site. These are Gly164, His197, Ser198, Asp199, Ser312, Pro315, Ile332, Glu335, Arg336, and Lys337. The image of the tertiary structure of CMAH is shown with the active pocket (Figure 2A), and Figure 2C shows the three-dimensional structure of the native ligand.

### 3.2. Analysis and Visualisation of the Docked Complex of CMAH and CMP-Neu5Ac

**Figure 3:**
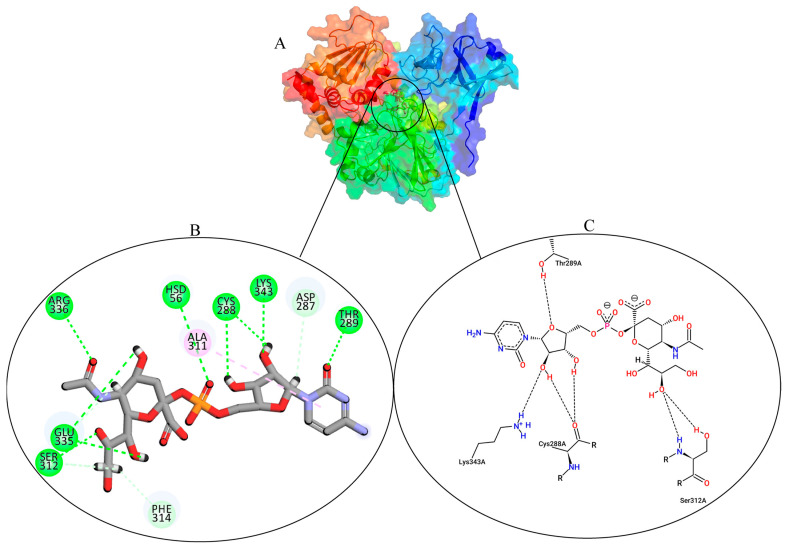
Protein plus image showing the native ligand inside the active pocket (**A**). Discovery studio analysis showing the different interactions. The dashed green lines indicate hydrogen bonds, and the magenta indicates hydrophobic interactions (**B**). Two-dimensional (2D) images of the interactions by protein plus (**C**).

The AutoDock Vina predicted the ideal pose with a Vina score of ‒8.7 kcal/mol. A strong druggability value was disclosed by the cross-validated DoGSiteScorer result, which also showed the native ligand’s binding pose with the CMAH (Figure 3A). The tool predicted a volume of 1052.2 Å^3^, a surface of 1105.62 Å^2^, a drug score of 0.8, and a simple score of 0.59. The Discovery studio image (Figure 3B) and protein plus (Figure 3C) show two-dimensional (2D) images of the interacting amino acid residues and non-covalent interactions.

### 3.3. Identification of Potential Inhibitors through Structure-Based Virtual Screening

**Figure 4:**
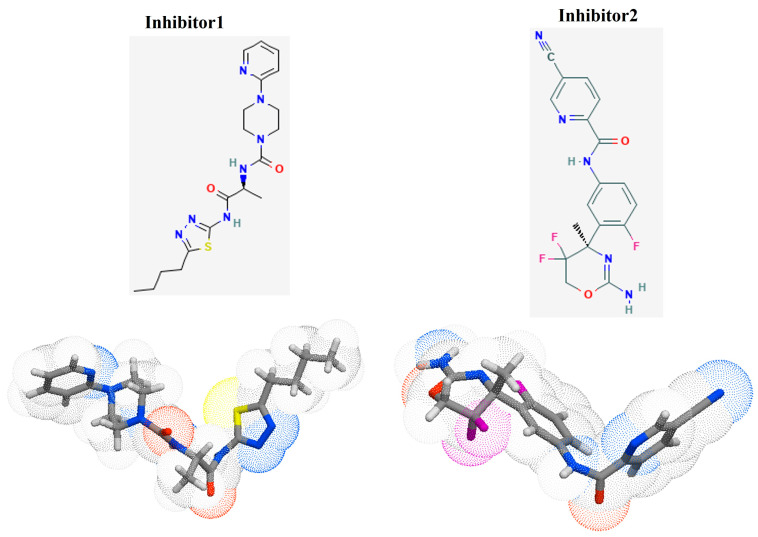
The 2D and 3D images of the inhibitor 1 (PubChem CID 52900436, N-[(2S)-1-[(5-butyl-1,3,4-thiadiazol-2-yl)amino]-1-oxopropan-2-yl]-4-pyridin-2-ylpiperazine-1-carboxamide) and inhibitor 2 (PubChem CID 53241828, N-[3-[(4r)-2-Azanylidene-5,5-Bis(Fluoranyl)-4-Methyl-1,3-Oxazinan-4-Yl]-4-Fluoranyl-Phenyl]-5-Cyano-Pyridine-2-Carboxamide), Adapted with permission from the PubChem database [44].

**Figure 5:**
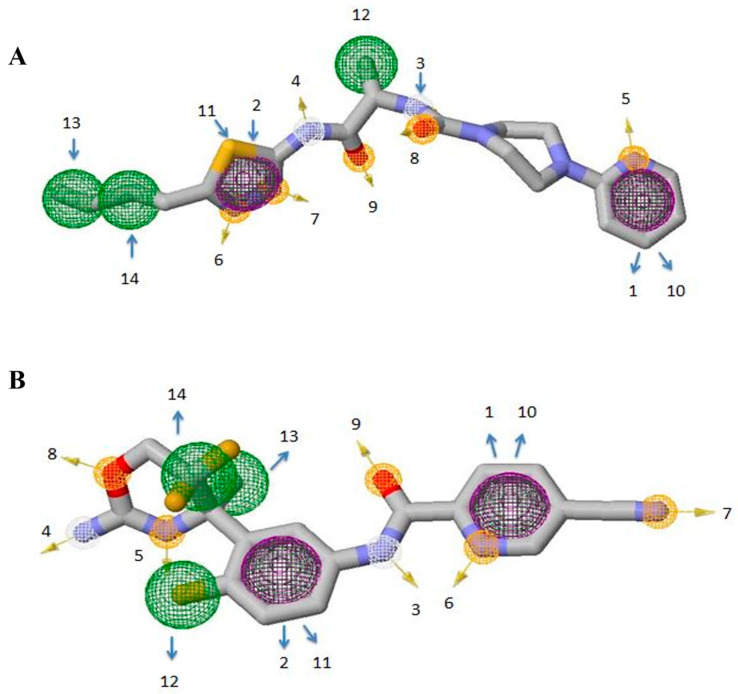
Depiction of pharmacophore models produced through ZINCpharmer. (**A**) Model of Inhibitor 1 and (**B**) Model of Inhibitor 2. Hydrophobic interactions are shown by green spheres; hydrogen acceptors are represented by orange spheres; hydrogen donors by white spheres, while aromatic rings are portrayed with purple spheres. The arrows pinpoint the relative positions of identified features.

In this study, the top two inhibitors, MCULE-5735538220-0-1 (Inhibitor 1) and MCULE-3985112460-0-5 (Inhibitor 2), as shown in Figure 4, were selected. The primary basis for their selection was based on having the best Vina docking score of −9.9 kcal/mol and −9.4 kcal/mol, respectively. In addition, physicochemical property analysis based on the parameters listed above and the different types of non-covalent interactions were also used as selection criteria. The selected inhibitors were further analysed for their pharmacophore properties. The pharmacophore models of inhibitor 1 and inhibitor 2 (Figure 5 and Tables S1 and S2) represent the relative binding affinities at different positions of the molecules forming the inhibitors.

The dimensions (x, y, and z) and the radius are also depicted (Tables S1 and S2). Each pharmacophore feature is highlighted with a specific colour and numbered to distinguish the classes. At two positions (10 and 11) hydrophobic atoms merged with aromatic rings, but overall, purple is displayed to highlight the features. In total, 14 positions that represent the distinct pharmacophore classes were identified.

**Figure 6:**
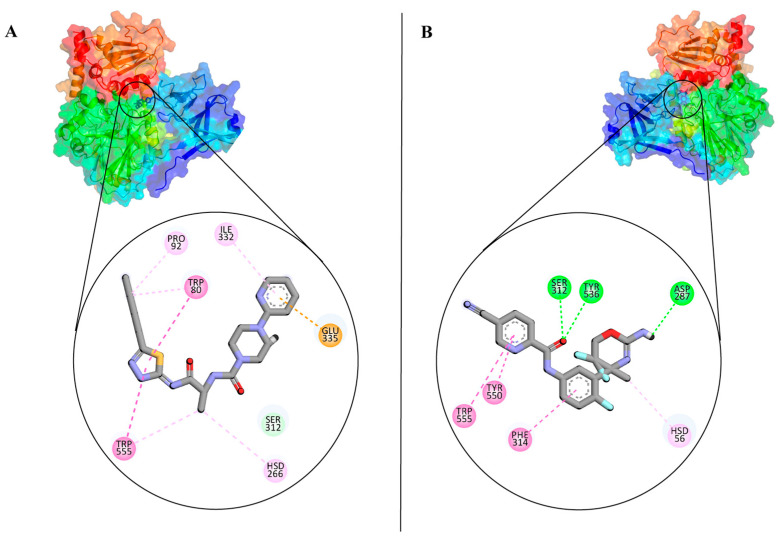
Protein plus images showing inhibitor 1 (**A**) and inhibitor 2 inside the active pockets (**B**). The 2D image of the inhibitors are also shown on the left side.

**Figure 7:**
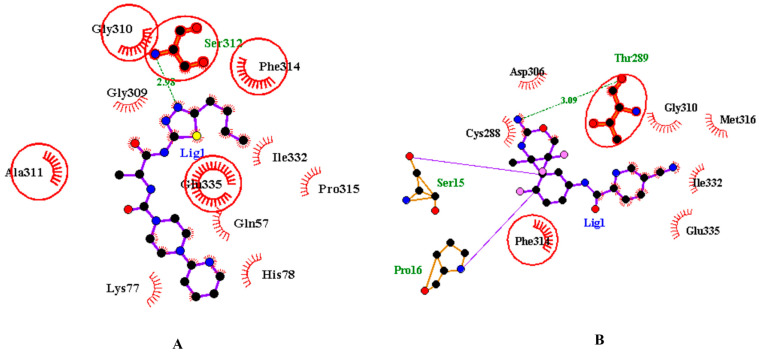
Two-dimensional representation of inhibitors docked with the catalytic domain of CMAH. (**A**) Inhibitor 1 docked with the catalytic domain and superimposed on apoprotein-CMAH and inhibitor 1 complex. (**B**) Inhibitor 2 docked with the catalytic domain and superimposed on apoprotein-CMAH and inhibitor 2 complex. Bold and circled residues represent common residues in the catalytic domain-inhibitor and apoprotein–CMAH–inhibitor complex.

The inhibitors were also analysed for their pharmacokinetic properties. Compared to the CMP-Neu5Ac, both inhibitors have a lower molecular weight, fewer numbers of hydrogen bond acceptors and donors, and rotatable bonds. Both inhibitors are favourable for drug-likeness analysis. The pharmacokinetic properties of inhibitors and CMP-Neu5Ac are provided in Table S3. Figure 6A,B show the inhibitors inside the active site pockets and 2D images of non-covalent interactions involved. To ensure the accuracy of molecular docking, the catalytic domain of CMAH was also docked with both inhibitors and superimposed with the apo-CMAH docked complex to identify similarities and differences. The analysis indicated that residues Gly310, Ala311, Phe314, and Glu335 interacted actively with inhibitor 1 in apo-CMAH-inhibitor 1 and catalytic domain-inhibitor 1 complexes. Similarly, Phe314 and Thr289 interacted in apoprotein-CMAH-inhibitor 2 and catalytic domain-inhibitor 2 complexes (Figure 7).

**Figure 8:**
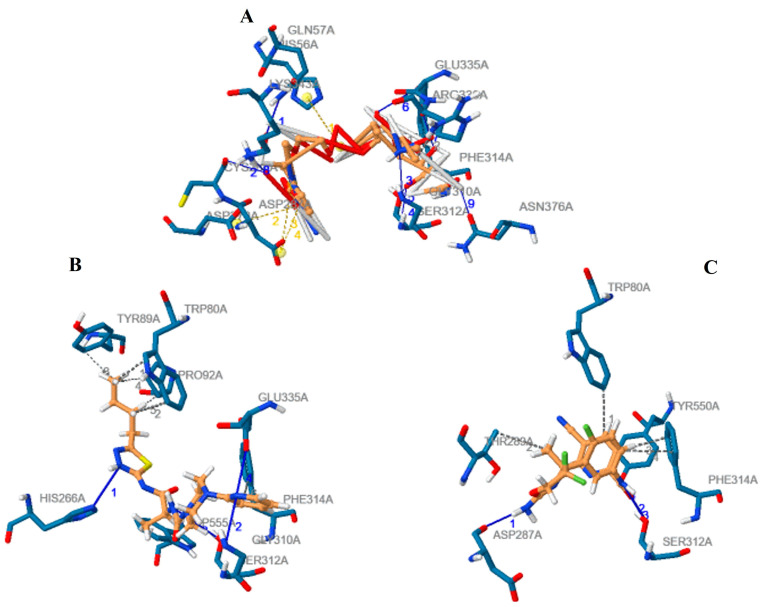
The detailed comparative assessment by the PLIP tool of the different interactions of the CMAH complexes with the native ligand (**A**), inhibitor 1 (**B**) and inhibitor 2. (**C**) The hydrophobic interactions, hydrogen bonds, and salt bridges are represented by the grey dash line, blue line, and yellow dash line, respectively.

We further performed a detailed comparative analysis among the three complexes (CMAH complexes with the native ligand and the two inhibitors) using the PLIP tool to identify all possible non-covalent interactions, such as hydrogen bonds, hydrophobic interactions, and salt bridges. Figure 8 shows different interactions of the three complexes, and Table 1 shows details of the amino acid residues involved. The amino acid residues Ser312 and Phe314 were common interacting amino acid residues among the three complexes.

### 3.4. Molecular Dynamic Simulations of the Complexes

**Figure 9:**
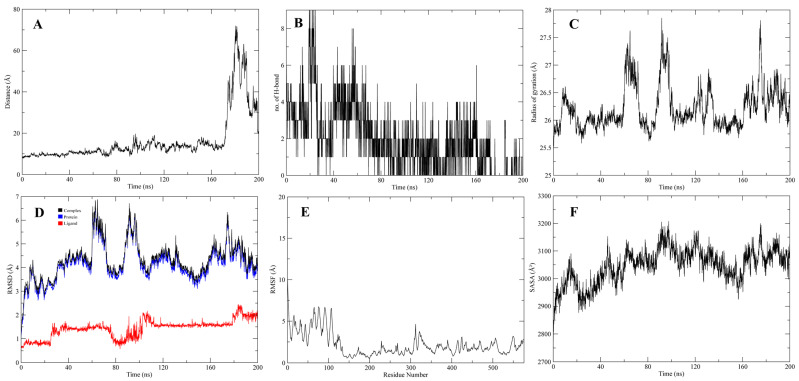
200 ns of MD Simulation analysis of CMAH–native ligand complex. (**A**) Average distance analysis. (**B**) Number of hydrogen bonds. (**C**) Rg, (**D**) RMSD, (**E**) RMSF, and (**F**) SASA.

**Figure 10:**
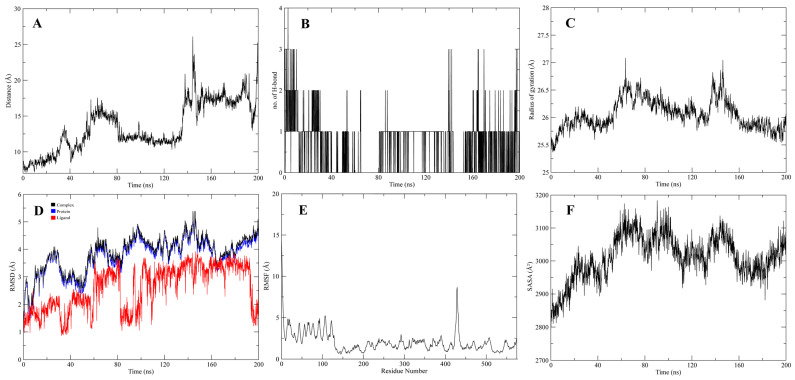
200 ns of MD Simulation analysis of CMAH-inhibitor 1. (**A**) Average distance analysis. (**B**) Number of hydrogen bonds. (**C**) Rg, (**D**) RMSD, (**E**) RMSF, and (**F**) SASA.

**Figure 11:**
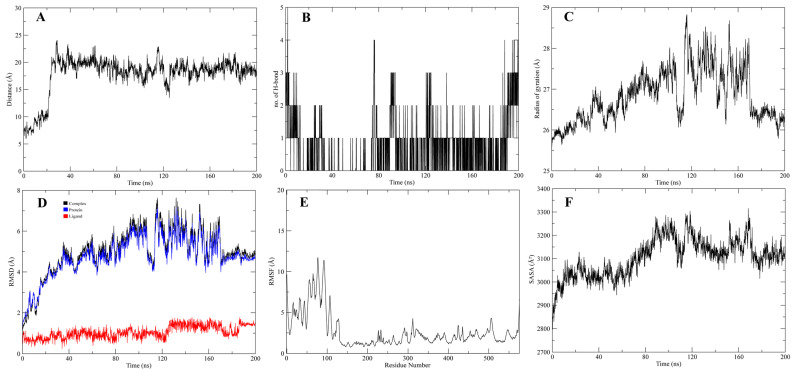
200 ns of MD Simulation analysis of CMAH-inhibitor 2. (**A**) Average distance analysis. (**B**) Number of hydrogen bonds. (**C**) Rg, (**D**) RMSD, (**E**) RMSF, and (**F**) SASA.

The stable interactions of the native ligand, inhibitors 1 and 2, with CMAH, were further evaluated through molecular dynamics (MD) simulations. Analyses related to Root Mean Square Deviation (RMSD), Root Mean Square Fluctuation (RMSF), surface accessibility (SASA), the Radius of gyration (Rg), the average distance between protein and ligand, and the number of hydrogen bonds were investigated in 200 ns simulation (Figure 9, Figure 10 and Figure 11). The analyses indicated that, after 60 ns of simulation, the native ligand (CMAH-CMP-Neu5Ac) complex shows fluctuations in its average distance, RMSD, and Rg values (Figure 9). CMAH-inhibitor 1 complex also shows increased average distance, RMSD, and Rg after 60 ns, but did not show that RMSD stabilises after 80 ns (Figure 10). Comparatively, the CMAH-inhibitor 2 complex shows a sudden increase in average distance after 20 ns, and then the motion stabilises and elevation in RMSD after 30 ns (Figure 11). In terms of hydrogen bond number, CMP-Neu5Ac made more hydrogen bonds with CMAH compared to inhibitors 1 and 2 at the beginning of the simulation. Still, the number of hydrogen bonds dropped after 80 ns duration. Compared to CMP-Neu5Ac, both inhibitors only made 2–4 hydrogen bonds throughout the simulation, but the interaction was maintained. Analysis based on simulation shows that CMP-Neu5Ac made strong interaction with CMAH, but RMSF, SASA, RMSD, Rg, and average distance values indicate fluctuations in the interaction after 60 ns. Compared to the native ligand, the inhibitors’ interaction with CMAH stabilised after the first 20 ns. Additionally, at the end of the 200 ns, both inhibitors showed a stabilised interaction compared to the native ligand.

**Figure 12:**
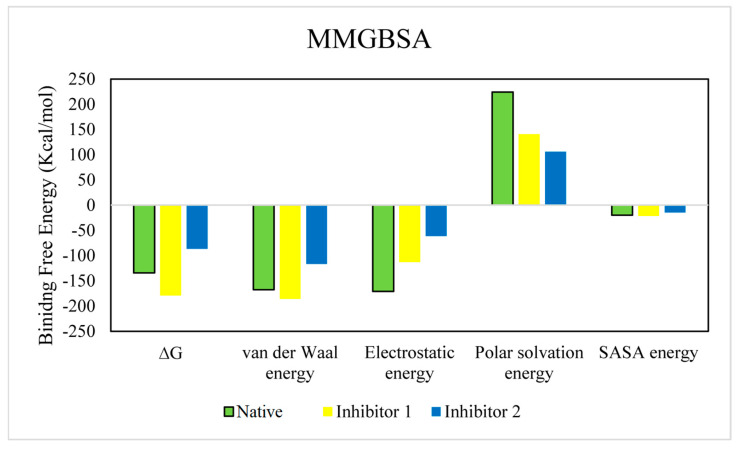
The comparison of binding free energy terms in the native and the identified inhibitors.

The relative binding free energies of the tested compounds were calculated by implying the MMGBSA module. The total binding free energy is the sum of electrostatic, van der Waals, Polar solvation, and SASA energy. The binding energy terms of the identified inhibitors were compared with that of the native ligand (Figure 12). The ∆G of the native ligand was −134.31 kcal/mol, while those of inhibitors 1 and 2 were −179.038 kcal/mol and −86.716 kcal/mol. This suggests that inhibitor 1 might have a stronger binding interaction [63]. The relative binding free energy values of the complexes are shown in Table 2.

## 4. Discussion

The Neu5Gc is a crucial sialic acid sugar molecule associated with pathogenic interactions in pig cells. With this considered, its role in xenoreactivities is linked to certain diseases (cancer, atherosclerosis, and rheumatoid arthritis are some of the inflammatory and autoimmune disorders), as described in the introduction. Our results highlight the need for more studies into the health impacts of Neu5Gc xenoreactivity and the development of measures to lessen the possible negative consequences [64]. Therefore, in this study, we employed a computational technique called computer-aided drug discovery (CADD) to identify novel inhibitors of the CMAH enzyme. 

Molecular docking was utilised in this investigation. The first step was to clarify the interactions between CMAH and the native ligand (CMP-Neu5Ac). The analysis of the CMAH–native ligand complex revealed that the enzyme established seven hydrogen bonds at the amino acid residues Gln57, Cys288, Gly310, Ser312, Glu335, Lys343, and Asn376. Hydrogen bonds facilitate protein–ligand interactions and are vital in protein folding catalysis [65]. In addition, two hydrophobic interactions were found between Phe314 and Arg336 amino acids. Hydrophobic interactions are weak intermolecular interactions crucial for stabilising energetically favoured ligands at the protein interface [66]. These interactions can enhance the binding efficiency of ligands or drugs [67]. When hydrophobic interactions are maximised, they also can impact drug side effects and toxicity [66].

Salt bridges are the most potent non-covalent molecular interactions [68] and can contribute to conformational specificity [69]. Similar to disulfide bonds, salt bridges can likewise serve as significant interactions [70]. Three salt bridges were detected at the amino acid sites: His56, Asp270, and Asp287. A Vina score of -8.7 kcal/ suggests that the CMAH could have a high affinity for the native ligand, as shown by the abundant evidence of interactions.

Enzyme inhibitors are low-molecular-weight chemical molecules that can inhibit or reduce enzymatic activities either irreversibly or reversibly [71]. This type of inhibitor binds non-covalently to the enzyme’s active site and competes with the native ligand. Reversible competitive inhibitors are among the common inhibitors employed in pharmaceutical industries to combat various diseases. For instance, polyoxins and nikkomycins are essential in treating fungal infections, functioning as competitive inhibitors to UDP-N-acetyl glucosamine, a substrate for chitin formation [72]. 

High-throughput virtual screening was employed to identify two novel competitive inhibitors based on their interactions and Vina scores. Top Vina scores (higher negativity) could indicate more efficient binding interactions [27,28,29,30,31]. Inhibitor 1, N-[(2S)-1-[(5-butyl-1,3,4-thiadiazol-2-yl)amino]-1(,N-[(2S)-1-[(5-butyl-1,3,4-thiadiazol-2-yl-1-oxopropan-2-yl]-4-pyridin-2-ylpiperazine-1-carboxamide) had a higher Vina score (−9.9 kcal/mol) than the native ligand. This might be an indication that this inhibitor may bind more efficiently than the native ligand; however, in the introduction, we highlighted the non-reliability of Vina scores [32,33]. The protein inhibitor 1 study revealed that this inhibitor interacted with CMAH and formed five hydrophobic contacts at the Trp80, Tyr89, Pro92, Phe314, and Trp555 amino acid residues.

Additionally, analysis of the complex uncovered four hydrogen bonds at the amino acid residues His266, Gly310, Ser312, and Glu335. Unlike that of the native ligand, no salt bridges were found. The native ligand and inhibitor 1 contain a hydrophobic interaction with the amino acid residue Phe314. Similar hydrogen bonds were found at the amino acid residues Gly310, Ser312, and Glu335 of both the native ligand and inhibitor 1. Therefore, these similarities may have contributed to the highest Vina score for inhibitor 1.

The second-best or most effective inhibitor (inhibitor 2) identified based on the Vina score was N-[3-[(4r)-2-Azanylidene-5,5-Bis(fluoranyl)-4-Methyl-1,3-Oxazinan-4-Yl]-4-Fluoranyl-Phenyl]-5-Cyano-Pyridine-2-Carboxamide. It also had a greater Vina score (−9.4 kcal/mol) than the native ligand. Although no salt bridges were discovered, four hydrophobic interactions and three hydrogen bonds were observed at the following amino acid residues: Trp80, Thr289, Phe314, Try550 and Asp287, Ser312, and Try550, respectively. Hydrophobic interaction and hydrogen bonds were discovered at the Phe314 and Ser312 amino acid residues, compared to the native ligand. When the three complexes were compared, phe314 and Ser312 were common amino acid residues involved in hydrophobic interactions and hydrogen bonding. Therefore, these amino acid residues are assumed to be crucial for the enzyme’s catalytic activity.

The two inhibitors obtained from virtual screening were further subjected to pharmacophore analysis to study the steric and electronic features of the inhibitors. The pharmacokinetic qualities of the inhibitors were also assessed. A compound may be affected by many different variables, including the drug’s chemical structure, formulation, method of administration, and interactions with other pharmaceuticals and physiological conditions [73]. Both inhibitors passed the drug-likeness investigation. Inhibitor 1 had lower water solubility than inhibitor 2. Overall, both inhibitors have bioactive properties and can be used for therapeutic purposes.

MD simulations were employed further to analyse the complexes. MD simulation analysis consisted of RMSD, RMSF, Rg, SASA, average distance, and hydrogen bond analysis. MD simulations are extensively performed in the pharmaceutical industry to facilitate drug discovery. The algorithms of MD simulations employ particle velocity and quantum mechanics to measure particle charges, bond energies, angles, and distances [74]. Through this information, an assessment of the structure–function relationship can be easily performed, facilitating drug discovery [75]. Interestingly, in this study, non-convergence was observed in the MD simulation analysis of protein–ligand complexes, which was obvious for the native ligand and the inhibitor 1. This can arise from various factors, among which is possible inherent protein flexibility, which could have impacted the convergence [76,77] and is associated with large conformational changes [76], multiple stable conformations [78], or intrinsically disordered regions [76]. Intrinsically disordered regions of the CMAH protein were also observed in our previous study on the predicted impacts of mutations on bovine CMAH [79].

## 5. Conclusions and Recommendations

Considering the importance of the Neu5Gc sugar molecule in the pathophysiology of pigs and its function in the development of several human diseases, it is imperative to identify the interacting partners, such as inhibitors, at the molecular level. Molecular structural studies of CMAH in complexes with its native ligand and two newly discovered inhibitors were performed using computational methods. Additionally, crucial enzymatic amino acid residues of the CMAH were predicted in addition to the active site. MD simulation analysis also determined the stable interaction between CMAH and the inhibitors. The study’s findings are a valuable contribution to the scientific literature. However, due to the possibility of intrinsically disordered regions of the protein, replica exchange molecular dynamics (REMD), metadynamics, or accelerated molecular dynamics (aMD), which can improve the exploration of conformational space and facilitate convergence [80], coupled with in vitro studies for determining the therapeutic potential, are advised for future studies.

## Figures and Tables

**Table 1:** The different amino acid residues involved in the various interactions.

Ligand	Molecular Formula	Hydrophobic Interactions	Hydrogen Bonds	Salt Bridges	Vina Score (Kcal/mol)
Native	C_20_H_31_N_4_O_16_P	Phe314 & Arg336	Gln57, Cys288, Gly310, Ser312, Glu335, Lys343, Asn376	His56, Asp270, Asp287	−8.7
Inhibitor 1	C_19_H_27_N_7_O_2_S	Trp80, Tyr89, Pro92, Phe314, Trp555	His266, Gly310, Ser312, Glu335	None	−9.9
Inhibitor 2	C_18_H_14_F_3_N_S_O_2_	Trp80, Thr289, Phe314, Try550	Asp287, Ser312, Tyr550	None	−9.4

**Table 2:** Calculated binding free energies of tested compounds (KJ/mol).

Compound	∆G	Van der Waal Energy	Electrostatic Energy	Polar Solvation Energy	SASA Energy
Native	−134.317	−167.815	−170.890	224.374	−19.985
	±89.180	±44.570	±162.416	±119.177	±3.904
Inhibitor 1	−179.038	−185.644	−112.857	140.880	−21.418
	±12.127	±13.649	±34.330	±46.563	±0.989
Inhibitor 2	−86.716	−116.820	−61.125	105.857	−14.629
	±22.650	±15.080	±48.771	±61.576	±2.232

## Data Availability

Not applicable.

## Supplementary Materials

The following supporting information can be downloaded at: https://www.mdpi.com/article/10.3390/pathogens12050684/s1, Table S1: Representation of pharmacophore class with positions, dimensions and radius of inhibitor 1; Table S2: Representation of pharmacophore class with positions, dimensions and radius of inhibitor 2; Table S3: The pharmacokinetic properties of inhibitors and CMP-Neu5Ac are provided i; Figure S1: Secondary structures of CMAH protein. Arrows depict the beta-sheets, and coiled structures show alpha folds. Different colours represent its different domains. The purple line indicates the N-terminal region and the orange line depicts the C-terminal region.
