# Supplementary material for: Molecular Structural Analysis of Porcine CMAH–Native Ligand Complex and High Throughput Virtual Screening to Identify Novel Inhibitors
Source: Pathogens. 2023 May 5;12(5):684. doi: 10.3390/pathogens12050684 (PMC10220980; doi:10.3390/pathogens12050684)
Supplement: Supplementary file 1 [file pathogens-12-00684-s001.zip › pathogens-2349206-supplementary.pdf]

SUPPLEMENTARY TABLE

Table S1: Representation of pharmacophore class with positions, dimensions and radius of inhibitor 1

Positions	Pharmacophore Class	x	y	z	Radius
1	Aromatic	7.55	−1.28	−0.33	1.1
2	Aromatic	−5.04	−0.53	0.05	1.1
3	Hydrogen Donor	0.48	1.86	−0.25	0.5
4	Hydrogen Donor	−2.99	1.1	0.14	0.5
5	Hydrogen Acceptor	7.38	0.1	−0.52	0.5
6	Hydrogen Acceptor	−5.37	-1.54	−0.51	0.5
7	Hydrogen Acceptor	−4.16	−0.9	−0.69	0.5
8	Hydrogen Acceptor	0.58	1.14	1.94	0.5
9	Hydrogen Acceptor	−1.41	0.2	−1.35	0.5
10	Hydrophobic	7.55	−1.28	−0.33	1
11	Hydrophobic	−5.04	−0.53	0.05	1
12	Hydrophobic	−1.18	3.41	−1.15	1
13	Hydrophobic	−10.49	−1.22	−0.34	1
14	Hydrophobic	−8.67	−1.24	0.22	1

Table S2: Representation of pharmacophore class with positions, dimensions and radius of inhibitor 2

Positions	Pharmacophore Class	x	y	z	Radius
1	Aromatic	5.03	0.33	−0.09	1.1
2	Aromatic	−1.09	−1.61	0.05	1.1
3	Hydrogen Donor	1.62	−0.96	0	0.5
4	Hydrogen Donor	−6.64	0	−1.32	0.5
5	Hydrogen Acceptor	−4.31	−0.06	−1	0.5
6	Hydrogen Acceptor	4.29	−0.86	0.09	0.5
7	Hydrogen Acceptor	9	0.19	0.11	0.5
8	Hydrogen Acceptor	−5.72	1.47	0.12	0.5
9	Hydrogen Acceptor	1.62	1.36	−0.4	0.5
10	Hydrophobic	5.03	0.33	−0.09	1
11	Hydrophobic	−1.09	−1.61	0.05	1
12	Hydrophobic	−3.75	−2.26	0.09	1
13	Hydrophobic	−2.57	1.56	−1.34	1
14	Hydrophobic	−3.24	0.98	1.47	1

Table S3. The pharmacokinetic properties of inhibitors and CMP-Neu5Ac.

Properties	CMP-Neu5Ac	Inhibitor 1	Inhibitor 2
Physicochemical Properties			
Formula	C20H30N4NaO16P	C19H27N7O2S	C18H14F3N5O2
Molecular weight	636.43 g/mol	417.53 g/mol	389.33 g/mol
Number. heavy atoms	42	29	28
Number. Aromatic. heavy atoms	6	11	12
Fraction Csp3	0.7	0.53	0.22
Number. rotatable bonds	12	10	4
Number. H-bond acceptors	17	5	8
Number. H-bond donors	9	2	2
Molar Refractivity	126.46	120.19	96.03
TPSA	335.55 Å^2	131.59 Å^2	113.39 Å^2
Lipophilicity			
Log Po/w (iLOGP)	−11.68	2.65	2.24
Log Po/w (XLOGP3)	−6.75	2.22	1.7
Log Po/w (WLOGP)	−5.15	1.18	3.12
Log Po/w (MLOGP)	−5.35	0.76	1.38
Log Po/w (SILICOS-IT)	−5.77	2.02	3.42
Consensus Log Po/w	−6.94	1.77	2.37
Water Solubility			
Log S (ESOL)	1.15	−3.45	−3.38
Solubility	9.05e+03 mg/mL ; 1.42e+01 mol/L	1.49e−01 mg/mL ; 3.56e-04 mol/L	1.63e-01 mg/mL ; 4.19e−04 mol/L
Class	Highly soluble	Soluble	Soluble
Pharmacokinetics			
GI absorption	Low	High	High
BBB permeant	No	No	No
P-gp substrate	Yes	Yes	Yes
CYP1A2 inhibitor	No	No	No
CYP2C19 inhibitor	No	Yes	Yes
CYP2C9 inhibitor	No	No	No
CYP2D6 inhibitor	No	No	No
CYP3A4 inhibitor	No	Yes	Yes
Log Kp (skin permeation)	−14.97 cm/s	−7.27 cm/s	−7.47 cm/s
Druglikeness			
Lipinski	No; 3 violations: MW > 500, NorO > 10, NHorOH > 5	Yes; 0 violation	Yes; 0 violation
Ghose	No; 3 violations: MW > 480, WLOGP < −0.4, #atoms > 70	Yes	Yes
Veber	No; 2 violations: Rotors > 10, TPSA > 140	Yes	Yes
Egan	No; 1 violation: TPSA > 131.6	Yes	Yes
Muegge	No; 5 violations: MW > 600, XLOGP3 < −2, TPSA > 150, H-acc > 10, H-don > 5	Yes	Yes
Bioavailability Score	0.11	0.55	0.55

SUPPLEMENTARY FIGURE

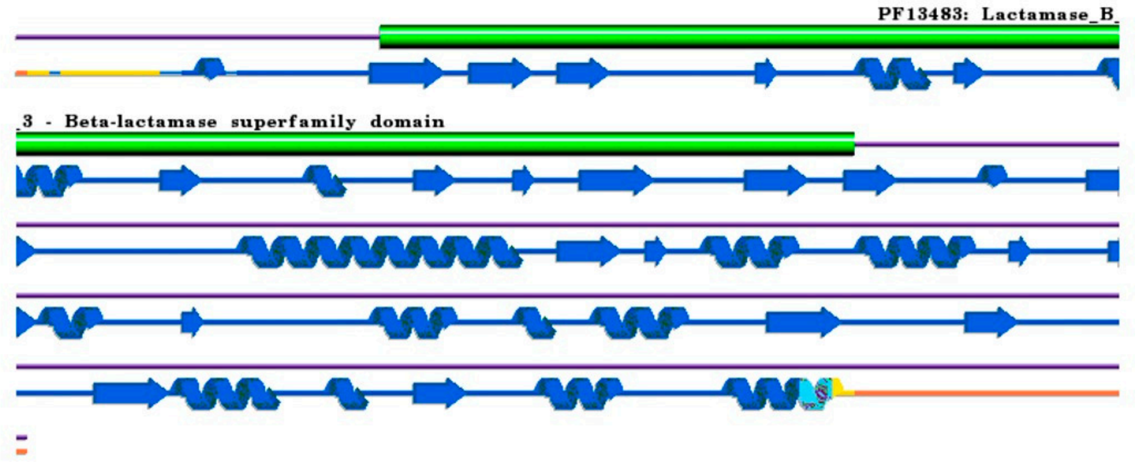

Figure S1: Secondary structures of CMAH protein. Arrows depict the beta-sheets, and coiled structures show alpha folds. Different colours represent its different domains. The purple line indicates the N-terminal region, and the orange line depicts the C-terminal region.
